# Gene therapy for haemophilia

**DOI:** 10.1111/j.1365-2141.2007.06942.x

**Published:** 2008-03

**Authors:** Samuel L Murphy, Katherine A High

**Affiliations:** Department of Pediatrics, Children's Hospital of Philadelphia Philadelphia, PA, USA

**Keywords:** haemophilia, gene therapy, vectors, adeno-associated virus

## Abstract

The ultimate goal of gene therapy is the replacement of a defective gene sequence with a corrected version to eliminate disease for the lifetime of the patient. This challenging task is not yet accomplished, however significant progress is evident. An initial spate of clinical trials attempting the treatment of haemophilia with gene transfer primarily resulted in the demonstration of good safety profiles, but without efficacy. Subsequent reengineering of vector plasmids and delivery systems resulted in markedly improved outcomes in animal models of the disease. The most recent clinical trial for the treatment of haemophilia B with gene transfer showed transient achievement of efficacy in the highest dose cohort tested, but also exposed a previously hidden barrier to the future success of these treatments. The progress and problems of gene therapies for haemorrhagic disorders will be discussed. This review will concentrate on approaches in or near clinical application.

## Overview of gene therapy

The ambitious objective of gene therapy is to edit a defective gene sequence *in situ* to achieve complete reversion of a disease phenotype for the lifetime of the patient. In spite of recent successes in site-specific correction of defective gene sequences, the focus of most gene therapy strategies to date is on gene addition rather than gene replacement (Urnov *et al*, 2005). This simplified approach relies on a delivery mechanism to provide a corrected copy of the defective gene without removal of the error-containing genomic sequence.

While literally hundreds of animal models of disease can now be effectively treated by gene transfer, a select few diseases remain the primary focus of much gene therapy research. A combination of factors including prevalence of disease, width of therapeutic window, ability to accommodate the corrected gene sequence in a gene transfer vector, reliability and availability of animal models of the disease, and funding and support from disease-specific foundations, all contribute to the overrepresentation of these few diseases.

Haemophilia A and B are among the most extensively researched diseases in the field of gene therapy. Small and large animal models of both diseases are available for preclinical testing. Importantly, treatment of the disease can be quantitatively measured through well-defined coagulation assays, eliminating a problem that plagues gene therapy efforts for many other disease entities. Another important aspect of the treatment of haemophilia by gene transfer is that there is a relatively low threshold for success. If long-term expression of the defective coagulation factor at 2–3% of wild-type levels could be achieved, then a substantial reduction in the clinical manifestations of the disease would be expected (Herzog *et al*, 1999; Sarkar *et al*, 2000). Expression of greater than 30% of the wild-type level of the defective coagulation factor would result in a phenotypically normal patient under most circumstances (Pollak and High, 2001), although higher levels may be required in the face of haemostatic challenge (Plug *et al*, 2006).

Another advantage of haemophilia B in the development of gene therapy strategies is the relatively small size of *F9* cDNA (∼1·4 kB of coding sequence). This is amenable to insertion into many different gene transfer vectors and allows the addition of numerous transcriptional regulatory elements to both improve and restrict transgene expression in select cell types. *F8* cDNA is much larger than that of *F9* (>8 kB), and is not as readily accommodated in gene transfer vectors. Several strategies have been employed to overcome this difficulty. A first step towards more efficient packaging of *F8* cDNA was deletion of the non-essential B-domain (Toole *et al*, 1986; Eaton *et al*, 1987). A dual-vector approach, in which the Factor VIII heavy chain and light chain are separately encoded by two different vector genomes, is another mechanism by which the large transgene can be accommodated (Mah *et al*, 2003). A third technique, one that also employs two vectors, fragments two halves of a cDNA and tethers them through the use of a 3′ splice donor site in vector genome A and a 5′ splice acceptor site in vector genome B (Yan *et al*, 2000; Lai *et al*, 2005). In the future, improvements in vector manufacturing may make it possible to package even the larger *F8* cDNA into the smaller gene transfer vectors without significantly compromising yield or homogeneity of vector preparations (Grieger & Samulski, 2005).

## Early clinical trials for gene transfer treatment of haemophilia

Between 1998 and 2001 five different Phase I clinical trials were initiated for the treatment of haemophilia by gene transfer (Kay *et al*, 2000; Roth *et al*, 2001; Manno *et al*, 2003; Powell *et al*, 2003). Several different gene delivery systems were used in these trials, including a retroviral vector, an adenoviral vector, two different adeno-associated viral vectors, and a non-viral gene delivery method. The results from each of these trials are discussed here with background on each of the vector delivery systems.

Retroviruses are RNA viruses which use reverse transcription to generate a double-stranded DNA intermediate during replication. Replication-defective retroviral vectors also contain an RNA genome that is reverse transcribed and integrated into the host genomic DNA. Integration provides the potential for long-term, persistent gene expression but also increases the risk of the treatment through the potential for insertional mutagenesis and/or insertional activation of proximal genes, as was observed following retroviral transduction of haematopoietic cells in a gene therapy trial for X-linked severe combined immunodeficiency (Hacein-Bey-Abina *et al*, 2003a,b; Fischer *et al*, 2004). Viral coding sequences are provided in *trans* during the manufacture of the vector and are not present at detectable levels in the vector itself. Many retroviruses, including murine leukaemia virus, are incapable of penetrating the nuclear membrane. These retroviruses and associated derivative viral vectors can only transduce dividing cells, requiring the natural breakdown of the nuclear membrane that occurs during cell division in order to enter the nucleus.

Given this limitation, it is not surprising that the most successful treatment strategies for liver-directed treatment of haemophilia using retroviral vectors devised strategies to induce hepatocyte cell division. One example of this was the use of a partial hepatectomy immediately preceding vector infusion (Kay *et al*, 1993). In this way, hepatocytes were induced to undergo cycling during the time of vector infusion. This approach only yielded low levels of Factor VIII expression (<1%), but partial correction for at least 5 months, in a canine model of haemophilia B. An alternative approach to target dividing hepatocytes is to infuse retroviral vectors into neonates, whose hepatocytes are naturally undergoing rapid cell division. This approach was successfully employed by VandenDriessche *et al* (1999) to fully correct Factor VIII deficiency in a murine model of haemophilia A. Using an identical treatment in 13 neonatal mice, 8/13 mice demonstrated greater than 50% of wild-type Factor VIII activity by COAtest (chromogenic Factor VIII activity test) assay. Long-term follow up revealed no significant loss of expression as long as 15 months after vector treatment. This approach was successfully extended to the canine model of haemophilia B by Xu *et al* (2003), who achieved up to 3·5% of normal Factor IX activity levels following retroviral vector transduction of neonatal haemophilia B dogs.

Previously conducted preclinical studies led to the initiation of a phase I clinical trial testing a Moloney murine leukaemia virus-based retroviral vector encoding B-domain deleted Factor VIII for the treatment of haemophilia A (Greengard & Jolly, 1999; Roehl *et al*, 2000; McCormack *et al*, 2001; Powell *et al*, 2003). Doses ranging from 2·7 × 10^7^ transducing units (TU)/kg to 4·4 × 10^8^ TU/kg were tested. The treatment was well tolerated by all subjects and replication competent virus was undetectable in all of the collected samples. Factor VIII levels above 1% were sporadically detected but did not correlate with the dose administered. Vector DNA was detectable in peripheral blood mononuclear cells (PBMCs) in 4/8 subjects that received 2·8 × 10^7^ TU/kg and 8/8 subjects that received 9·2 × 10^7^ TU/kg up to 53 weeks after the infusion. Overall, the signs of clinical improvement following vector infusion were modest at best. These findings were consistent with animal studies suggesting that efficient retroviral transduction of hepatocytes would require higher doses and some degree of mitotic induction.

Adenoviral vectors have been frequently used in preclinical gene transfer studies of haemophilia. Advantages of these double-stranded DNA vectors are high transgene expression levels and the ability to transduce hepatocytes efficiently *in vivo*. Frequently, however, expression from early generation vectors was transient, owing to the immunogenic properties of the adenoviral vectors themselves. In contrast to other vectors that are devoid of viral gene sequences, early generation adenoviral vectors encode many viral proteins in addition to the transgene; these may contribute to the immunogenicity of these vectors (Schagen *et al*, 2004). In mice, the immune response was not sufficient to ablate the effects of the treatment and curative levels of Factor VIII or Factor IX could be achieved and sustained for 3–5 months in murine models of Haemophilia A and B (Smith *et al*, 1993; Connelly *et al*, 1996; Walter *et al*, 1996). In contrast, large animal studies demonstrated that the duration of transgene expression was significantly shorter than that seen in mice and that hepatotoxicity was correspondingly greater (Kay *et al*, 1994; Lozier *et al*, 1999). In a canine study, a vector dose of 2·4 × 10^12^ plaque forming units (pfu) infused into three haemophilia B dogs resulted in transient expression of Factor IX at supraphysiological levels. These levels rapidly declined, reaching 1% of normal by 3 weeks after vector infusion and 0·1% 2 months postvector infusion. A comparable dose of the same vector in mice resulted in sustained levels of Factor IX expression at 20% of normal for over 4 months (Kay *et al*, 1994). Further substantiating these findings was a non-human primate study in which an adenovirus vector was used to deliver a human Factor IX transgene. In this study, a peak of 80% wild-type Factor IX levels was achieved at the highest dose (1 × 10^11^ pfu/kg) followed by a rapid decline to baseline, with human Factor IX levels undetectable by 3 weeks after vector infusion (Lozier *et al*, 1999). Moreover, significant hepatotoxicity was observed. Serum aspartate transaminase (AST) levels rose above 500 U/ml at both the intermediate dose and the high dose, and bilirubin levels rose above 85·5 μmol/l in the high dose cohort only (Lozier *et al*, 1999). Thrombocytopenia was also observed in the intermediate dose and high dose cohorts. Thrombocytopenia was subsequently linked to decreased fibrinogen levels and increased platelet clearance time believed to result from the hepatotoxicity of the vector (Lozier *et al*, 1999; Wolins *et al*, 2003). Recent work by Othman *et al* (2007) showed that activation of platelets occurs following exposure to adenovirus. Activation is, in turn, followed by upregulation of P-selectin, which is known to promote platelet clearance (Othman *et al*, 2007). They also identified a critical role for von Willebrand factor (VWF) in the induction of thrombocytopenia following adenovirus infusion. Using a vector dose that results in thrombocytopenia in wild-type mice (1 × 10^11^ pfu), significant thrombocytopenia was not observed in VWF knockout mice.

Further development of adenoviral vector production systems allowed complete elimination of viral coding sequences from the vector. The intent of this manoeuvre was to minimize the immunogenicity of these vectors. Using these ‘gutted’ adenoviral vectors, sustained correction of disease phenotype was observed in haemophilia A mice infused with 3 × 10^12^ vector particles (vp)/kg with no apparent hepatotoxicity. Non-human primate studies showed some dose-dependent hepatotoxicity with the threshold residing between 1·4 × 10^12^ vp/kg and 4·3 × 10^12^ vp/kg. A Phase I clinical trial was initiated to test the safety of an adenoviral vector encoding human Factor VIII for the treatment of Haemophilia A; because of *a priori* concerns regarding immunogenicity, the trial was structured to monitor carefully for changes in liver function tests or platelet count. The first subject, enrolled at the lowest dose of 4·3 × 10^10^ vp/kg, experienced inflammation, fever and myalgia upon vector infusion; these symptoms are commonly observed with infusion of adenoviral vectors. The subject also experienced thrombocytopenia and an elevation in serum transaminases that peaked 7 d after infusion and returned to baseline by 19 d postinfusion (Chuah *et al*, 2004). Due to safety concerns and a perceived narrow therapeutic index, no additional subjects were enrolled in this trial.

A third vector modality used in this first group of clinical trials was the adeno-associated virus (AAV) vector. AAV is a small, single-stranded DNA virus that is naturally replication-defective. It relies on functions of the gene products of helper viruses, such as adenovirus or herpesvirus, in order to complete its replication cycle. There are no known symptoms or diseases associated with AAV infection. The small vector genome results in a small coding sequence capacity for AAV vectors, making construction of *F9* vectors more straightforward than construction of those for Factor VIII (although studies employing AAV vectors for the treatment of animal models of haemophilia A have been successful). Despite the fact that the vector DNA remains largely episomal (i.e. without vector genome integration), AAV vectors can nonetheless direct long-term expression of a transgene, if introduced into a long-lived, postmitotic (non-dividing) target cell (Nakai *et al*, 2001; Song *et al*, 2004). Early work with AAV vectors for the treatment of haemophilia B used muscle-targeted vectors encoding the Factor IX transgene under the control of the cytomegalovirus (CMV) promoter. With this construct, therapeutic levels of serum Factor IX (200–350 ng/ml) were achieved in mice (Herzog *et al*, 1997). Unlike other viral vectors, transduction of muscle cells with AAV vectors resulted in sustained expression of the transgene in immunocompetent mice, even when a foreign transgene such as beta-galactosidase was used (Xiao *et al*, 1996; Fisher *et al*, 1997; Monahan *et al*, 1998). Muscle-directed AAV vectors were subsequently shown to achieve low but possibly clinically relevant plasma levels (70 ng/ml) of canine Factor IX in haemophilia B dogs at a vector dose of 8·5 × 10^12^ vp/kg. This level of expression was sustained for greater than 17 months (Herzog *et al*, 1999).

In 1999, a Phase I/II clinical trial was initiated to test the safety and efficacy of intramuscularly delivered AAV vectors encoding a CMV-driven *F9* transgene for the treatment of haemophilia B. Doses ranged from 2 × 10^11^ vector genomes (vg)/kg to 1·8 × 10^12^ vg/kg. The vector infusion was well tolerated in all subjects, with no adverse events related to vector infusion. No toxicity has been observed in over 7 years of follow-up after vector administration (Kay *et al*, 2000; Manno *et al*, 2003). Importantly, transgene expression was evident and sustained. Muscle biopsies taken 2 months, 10 months and >3 years after vector administration revealed the persistence of vector genomes as determined by Southern blot as well as local expression of Factor IX protein as shown by immunofluorescent staining (Manno *et al*, 2003; Jiang *et al*, 2006). This was strong evidence that the animal models of AAV vector-mediated gene transfer were predictive of the outcome in humans and that sustained transgene expression was possible. The barrier to the use of this approach as a treatment for haemophilia was the number of muscle injections that would be required in an adult haemophilia patient. Cell culture and animal studies previously established a limit to the amount of correctly processed, functionally active Factor IX protein that could be produced in muscle cells (Arruda *et al*, 2001; Herzog *et al*, 2002). This stipulated a requirement for a larger number of muscle cells to be transduced by a larger vector dose, rather than the transduction of the same number of muscle cells with larger doses of vector. Accordingly, more intramuscular injections of the vector would be required, and the number of injections thought to be required to reach a therapeutic effect was deemed clinically impractical.

In addition to viral vector delivery methods, one non-viral delivery method was also tested in a clinical trial (Roth *et al*, 2001). This approach, similar in some respects to previous clinical studies conducted in China (Lu *et al*, 1993; Qiu *et al*, 1996), consisted of transplantation of *F8*-transduced autologous fibroblasts. After isolation from a skin biopsy, patient cells were transfected with a plasmid encoding a human *F8* cDNA *ex vivo* and stable selection for transfectants was carried out. Single clones were expanded and tested for Factor VIII expression level, as well as tumorigenicity and microbial safety, prior to reimplantation onto the omentum. Animal model studies were promising for this approach, but data acquired from the Phase I clinical trial showed only a modest and temporary indication of positive effects. The treatment was, however, well tolerated and leaves open the possibility of future attempts using more potent expression systems for the *ex vivo* transduction and selection process. An important step in advancing this treatment modality will be the determination of the cause for the apparent loss of expression over time. Possible obstacles to durable transgene expression include: senescence of the implanted cells, promoter inactivation, fibrosis around the transplanted cells and immune responses to the gene-modified cells.

## Recent developments in gene transfer for haemophilia

Adenovirus vectors are still being pursued as a means of obtaining long-term expression of both Factor VIII and Factor IX. Improved liver-specific promoters and further redesign of production methods resulted in long-term expression of Factor VIII in canine models of haemophilia A, although some hepatotoxicity remains evident and could complicate translation into clinical trials (Andrews *et al*, 2002; Chuah *et al*, 2003; Brown *et al*, 2004). Specifically, inter-subject variation and a small therapeutic index make the safe use of adenoviral vectors difficult for stable gene transfer in humans. As a result of their inherent immunogenicity, adenoviral vectors are now more frequently used as vaccine delivery vehicles (Tatsis & Ertl, 2004).

New developments in the field of retroviral vectors are more promising for application in the treatment of haematological disorders. One of the most important innovations has been the development of lentiviral vectors, which have several advantages over the first-generation retroviral vectors. First, they are capable of transducing non-dividing cells, making them more suitable for transduction of, for example, hepatocytes and haematopoietic stem cells. Second, while retroviral vectors preferentially integrate their genomes near transcriptional start sites, lentiviral vectors show a random integration pattern into the open-reading frames of genes (Mitchell *et al*, 2004). While this difference does not eliminate the risk of insertional mutagenesis, it seems likely to mitigate the risk by reducing the number of full length gene transcripts that might be activated through vector genome insertion. Improvements in insulator elements flanking coding sequences within the vector genome itself further reduced the potential for undesirable insertional activation events (Chung *et al*, 1997). Naldini *et al* showed that the use of a liver-specific promoter in place of a CMV promoter could alone be a determinant of stable lentiviral transduction of hepatocytes (Follenzi *et al*, 2004). With a ubiquitous CMV promoter driving expression of either green fluorescent protein (GFP) or human Factor IX, expression was short-lived and the loss of expression was accompanied by both antibody formation against the transgene and T cell infiltrates in the liver. In contrast, use of a liver-specific promoter resulted in long-term, stable expression of GFP or human Factor IX in wild-type mice with expression levels of the latter reaching 200 ng/ml. In a subsequent study, Naldini *et al* also showed that the incorporation of four copies of a microRNA target sequence in the vector genome could selectively mark vector transcripts for destruction in cells expressing the corresponding microRNA (Brown *et al*, 2006). Using this lineage-specific suppression strategy, selective downregulation of transgene expression in haematopoietic cells was achieved. This vector demonstrated decreased immunogenicity and more robust, stable transgene expression in immunocompetent mice.

Silencing elements encoded by a lentiviral vector transcript were also used to demonstrate therapeutic effect in a recent study of lentiviral-mediated gene transfer for the treatment of sickle-cell disease. Unlike many other genetic disorders, sickle cell disease cannot be completely corrected by the addition of a wild-type copy of the defective gene. The mutant globin would act as a dominant negative gene product and suppress the activity of the wild-type therapeutic globin. Similar to the approach taken by Brown *et al* (2006), Samakoglu *et al* (2006) developed a small interfering RNA (siRNA) element targeting sickle-globin RNA that was inactive against the vector-encoded therapeutic gamma-globin transgene RNA. The siRNA was incorporated into an intron within the gamma-globin transgene. Following transcription of the transgene, both expression of the therapeutic gamma-globin and post-transcriptional downregulation of the beta-globin (sickle) RNA levels were observed. This experiment was conducted in haematopoietic cells taken from human patients with sickle cell disease, and provides proof-of-principle that this approach could be translated into the clinic. Chang *et al* (2006) recently demonstrated the potential for lentiviral transduction of haematopoietic stem cells for the production of Factor IX for the treatment of haemophilia B.

Continued advances were also made in animal models of AAV vector-mediated gene transfer for treatment of haemophilia. Substantial improvements in transgene expression levels were made through the use of liver-specific promoters and portal vein administration. Therapeutic levels of human Factor IX could be expressed and sustained in mice, dogs and non-human primates (Snyder *et al*, 1997; Wang *et al*, 1999; Mount *et al*, 2002; Nathwani *et al*, 2002). In wild-type mice, supraphysiological levels of 6·25 μg/ml were attained in haemophilia B mice from an AAV dose of 5·6 × 10^11^ vg/mouse (∼2 × 10^13^ vg/kg) (Wang *et al*, 1999). These levels were sustained without any apparent signs of diminution for over 20 weeks. Importantly, these results were scalable to larger animal models. Haemophilia B canines treated with a vector dose of 1–2 × 10^12^ vg/kg responded with expression of circulating canine Factor IX between 220 ng/ml and 590 ng/ml (4–12%) (Mount *et al*, 2002). Non-human primates infused through the portal vein with an AAV vector dose of 4 × 10^12^ vg/kg achieved human Factor IX levels of greater than 400 ng/ml (8%) which were sustained for more than 1 year (Nathwani *et al*, 2002). None of these studies reported liver toxicities or abnormalities in serum chemistries.

Further excitement was generated by the discovery of new AAV serotypes that were isolated from non-human primate tissues by polymerase chain reaction, using primers designed within known conserved sequences from previously identified AAVs (Gao *et al*, 2002). These new serotypes possessed several advantages over the more extensively studied, prototypical human-derived AAV2 serotype. AAV vectors are highly sensitive to antibody-mediated neutralization *in vivo* (Scallan *et al*, 2006). The frequency of antibodies to these novel AAV serotypes in the human population was greatly reduced compared to the frequency of the population carrying antibodies to the human derived strains of AAV (Gao *et al*, 2002; Scallan *et al*, 2006). This would mean the exclusion of fewer patients should a treatment ultimately be developed. Another advantage of the new serotypes is that, unlike AAV2 which must be delivered directly into the portal vein to achieve maximal liver transduction, the novel AAV8 serotype demonstrates equally efficient transduction of the liver in animals whether infused directly into the portal vein or into a peripheral vein. This finding was initially documented in mice and has been confirmed in subsequent non-human primate studies (Sarkar *et al*, 2004; Davidoff *et al*, 2005; Nakai *et al*, 2005). This provides a less invasive delivery method for clinical application. Yet another advantage seen with the novel AAV8 serotypes was far superior transduction efficiency in the liver, resulting in 10–100× greater transgene expression in mice (Gao *et al*, 2002; Nakai *et al*, 2005). In contrast to the scalability of route of administration, however, this finding did not ascend the evolutionary ladder. Non-human primate studies directly comparing multiple serotypes showed an attenuated advantage of the novel serotypes over other serotypes in stable transduction of hepatocytes (Davidoff *et al*, 2005; Gao *et al*, 2006). To date, neither AAV2 nor any of the novel serotypes have exhibited any immunogenicity or hepatotoxicity in animals, either at the time of administration or at later times. In fact, AAV vector transduction in the liver can result in the induction of immune tolerance to the transgene product (Mingozzi *et al*, 2003). This tolerance was sustained even after a strong challenge with an adenovirus vector expressing the identical transgene product.

The promise of animal studies using liver-directed AAV vectors prompted a second clinical trial testing the safety and efficacy of an AAV vector for the treatment of haemophilia B (Manno *et al*, 2006). In this trial, a liver-specific promoter was used to drive expression of the *F9* transgene. Vector was infused into the hepatic artery of haemophilia B subjects at three different doses: 8 × 10^10^ vg/kg, 4 × 10^11^ vg/kg and 2 × 10^12^ vg/kg. At the low and intermediate doses, no Factor IX expression was detected during the course of study. At the highest dose tested, 1/2 subjects (subject E) experienced a rise in Factor IX activity levels from an undetectable level at baseline to a peak of 11·2% 2 weeks after vector infusion. This level of Factor IX activity was accurately predicted by the preclinical animal studies (Mount *et al*, 2002). Four weeks after vector infusion, Factor IX activity levels remained above 10%, but at this time AST/alanine transaminase (ALT) levels rose above 100 IU/ml from a baseline of less then 50 IU/ml. By 5 weeks after vector infusion the Factor IX activity level declined to 6·1% and the AST/ALT levels increased to >200 IU/ml and >500 IU/ml, respectively. From this peak of transaminase activity, levels slowly declined to reach baseline 14 weeks postinfusion. Meanwhile, Factor IX activity level also receded to baseline undetectable levels by 10 weeks postinfusion.

This unexpected sequence of events prompted a thorough assessment of possible causes for the loss of transgene expression. A complete workup of the subject was able to rule out any adventitious infections (e.g. hepatitis B, CMV infection, etc). No antibody to Factor IX protein was detectable. One possible explanation for the loss of transgene expression was a T-cell mediated response to the transduced cells. This phenomenon had not been observed in any animal model of AAV transduction, but unlike the animal models, humans are frequently exposed to wild-type AAV viruses in the context of pathogens from very early childhood (Blacklow *et al*, 1968, 1971). These exposures, because they occur in the context of helper viruses, such as adenovirus that evoke a strong immune response, may also evoke an immune response to AAV vector proteins that could be reactivated by AAV vector infusion. Without serial PBMC collection, this hypothesis was difficult to test, and with the observation confined to a single subject, the chance of a repeat occurrence of this sequence of events was unclear.

Consequently, another subject (subject G) was enrolled at the intermediate dose (4 × 10^11^ vg/kg) with a PBMC collection protocol included to allow assessment of T cell responses to Factor IX and to AAV. The results of this analysis convincingly demonstrated that a T-cell response to the AAV vector capsid protein occurred following vector administration. The subject's T-cell responses were monitored during this time by interferon-gamma enzyme-linked immunosorbent assay (ELISpot), using pools of 15 amino acid peptides overlapping by 10 amino acids that spanned the entire vector capsid sequence and the entire *F9* sequence. Although T-cell responses to the vector capsid were undetectable at baseline, responses significantly above background were readily detected by the first PBMC collection time point, 2 weeks after vector infusion, and remained positive at each time point tested up to 12 weeks after vector infusion. Simultaneous ELISpot assays to detect immune responses to the Factor IX protein were consistently negative. In the 14 weeks following vector administration, subject G experienced a mild transaminitis (peak AST and ALT levels of 67 IU/l and 105 IU/l respectively) with a temporal course closely matched to that experienced by subject E (Manno *et al*, 2006). As with the other two subjects infused at this dose, no Factor IX activity levels >1% were detected following infusion of the vector. These findings strongly implicate a T cell response to AAV capsid in the loss of Factor IX expression in the subject who transiently expressed therapeutic levels of the transgene.

Epitope mapping using the results from the ELISpot assays identified a nine amino acid peptide that appeared to be an immunodominant epitope in the anti-capsid T-cell response of subject G. This knowledge was used to generate a pentamer reagent that enabled direct detection of the capsid-specific CD8^+^ T-cell population responding to this epitope. The kinetics of the T-cell response in subject G were mapped with this reagent and shown to overlap with the peak of serum transaminases (Mingozzi *et al*, 2007). Two years after vector infusion, PBMCs were isolated from subject E. No T-cell response to AAV capsid was detectable directly *ex vivo*. Upon expansion in the presence of AAV capsid peptides, however, a single immunodominant epitope from the AAV capsid invoked a significant expansion of T cells from the PBMCs of subject E. Following two rounds of PBMC expansion in the presence of this peptide, 8·8% of the CD8^+^ T cells in this culture secreted interferon gamma upon re-exposure to the epitope (Mingozzi *et al*, 2007). Also of note from these studies, immune responses to AAV capsid were detectable in normal, uninfused subjects in both PBMCs and splenocytes, suggesting that the events seen in this trial were not aberrant and would be repeated if additional donors were infused.

Given the likelihood that an immune response to AAV capsid ablated the transduced cells, and that capsid is present only transiently before being degraded and cleared from the cells, one potential solution is to block the immune response to capsid pharmacologically until capsid has been metabolized and cleared from the cells. This raises the question of the duration of persistence of the capsid in the transduced cell, or more precisely, the duration of persistence of peptide-major histocompatibility complexes on the cell surface. Attempts to generate an animal model that replicates these findings have so far been unsuccessful (Li *et al*, 2007a,b; Wang *et al*, 2007). Most approaches have relied on a prime-boost regimen with adenoviral or plasmid vectors expressing AAV capsid, to generate a robust CD8^+^ T cell response to AAV capsid. However, when these immunized mice are challenged with an AAV vector encoding a human *F9* transgene under the control of a liver-specific promoter (Li *et al*, 2007b), the transduced hepatocytes persist even in the presence of AAV capsid-specific T-cells. In fact, no diminution in transgene expression was detected as compared to unimmunized mice or control immunized mice.

## The next phase in gene transfer for haemophilia

Safe, long-term expression of clotting factors has been successfully achieved in large animal models of haemophilia using multiple gene transfer strategies, but these findings have not yet been translated into success in patients. Ongoing and proposed clinical studies should help to determine whether AAV-mediated gene transfer to the liver can achieve success. The initial liver trial is set to resume, at a dose approximately half that used in subject E, and with the addition of transient immunosuppression to block the T-cell response to the capsid. If a capsid-specific T-cell response was indeed causative in the loss of transduced hepatocytes in subject E, an important question remains: will the immunosuppressive regimen selected be sufficient to block the response? It is also not entirely clear how long the regimen will need to be maintained. Alternative hypotheses to explain the findings in the liver-directed AAV trial have been proposed. One hypothesis suggests that fragments of the AAV capsid were expressed in the transduced cells as a result of low-level packaging of these sequences during the vector production process (http://www4.od.nih.gov/oba/RAC/meeting.html). This hypothesis does not adequately explain the difference in findings between animal models and humans. A second hypothesis is that an alternate open reading frame contained within the *F9* cDNA encodes an immunogenic protein sequence, but this hypothesis also fails to explain the difference between animals and humans (http://www4.od.nih.gov/oba/RAC/meeting.html). Other hypotheses focus on differences in the sensitivity of immune responses in animals and humans. There is some evidence that T cells in humans may be more sensitive than T cells in non-human primates as a result of differential expression of an immunomodulatory lectin (Nguyen *et al*, 2006). Yet another possibility is that a low level of CpG methylated DNA is packaged into the vector during production and that humans possess more sensitive innate immune responses to these stimuli. Finally it has been proposed that there will be differences in capsid processing and presentation with alternate AAV serotypes, because of different kinetics of uncoating, differences in intracellular trafficking, or differences in antigen processing (Vandenberghe *et al*, 2006). Based on strong preclinical data in non-human primates, a proposed trial of AAV8 encoding a *F9* transgene will test this hypothesis (Nathwani *et al*, 2006). This next-generation vector carries a self-complementary expression cassette to enhance expression at lower doses and encodes a codon-optimized transgene to improve translational efficiency. Will the muscle ultimately be a better target for AAV-mediated Factor IX expression? It is clear that Factor IX transgene expression persisted in subjects injected with AAV vectors intramuscularly. New systemic approaches to introduce AAV vector into skeletal muscle have since been developed (Arruda *et al*, 2005). Will these delivery methods result in stable expression or transient expression of the transgene? Continuing studies should provide answers to these questions, and ultimately a safe and effective long-term treatment for haemophilia.
